# The General Public’s Perceptions of How the COVID-19 Pandemic Has Impacted the Elderly and Individuals with Intellectual Disabilities

**DOI:** 10.3390/ijerph19052855

**Published:** 2022-03-01

**Authors:** Maria R. Dekker, Noud Frielink, Alexander H. C. Hendriks, Petri J. C. M. Embregts

**Affiliations:** 1 Tranzo, Tilburg School of Social and Behavioural Sciences, Tilburg University, 5037 AB Tilburg, The Netherlands; mariaroos.dekker@gmail.com (M.R.D.); n.frielink@tilburguniversity.edu (N.F.); lex.hendriks@ru.nl (A.H.C.H.); 2 School of Pedagogical and Educational Sciences, Faculty of Social Sciences, Radboud University Nijmegen, 6525 XZ Nijmegen, The Netherlands

**Keywords:** elderly, intellectual disability, perceived impact, COVID-19, pandemic

## Abstract

This study examined the general public’s perceptions of how the COVID-19 pandemic has impacted the elderly and people with intellectual disabilities as well how these perceptions relate to people’s level of familiarity and contact quality with these groups. A cross-sectional survey was administered to a sample of the Dutch population (*n* = 1458 and *n* = 1761, comprising questions related to the elderly and people with intellectual disabilities, respectively). The general public was found to be generally aware of the deleterious impact of the pandemic upon the elderly and people with intellectual disabilities. Specifically, the respondents reported that both groups’ quality of life, physical and mental health, and quality and frequency of social contact was lower than it was prior to COVID-19, in addition to perceiving them as lonelier and less self-reliant. Notably, the impact on the elderly was considered to be greater than that on people with intellectual disabilities. Furthermore, those who had no familiarity with people with intellectual disabilities in real life perceived the impact to be lower than those who had a greater degree of familiarity. These findings have important implications, both for increasing awareness of the pandemic’s negative impact on these vulnerable groups and in terms of sufficiently addressing their specific needs and concerns. The findings also underscore that, particularly during the COVID-19 pandemic, it is important to increase the visibility of groups who already relied more on help and support from others in society prior to the pandemic, such as the elderly and people with intellectual disabilities, via, among other things, self-advocacy, education, and enhanced intergroup contact, in order to be able to sufficiently address their needs during these challenging times.

## 1. Introduction

The COVID-19 pandemic has had a substantial global impact. At the time of writing, there were over 262 million confirmed global COVID-19 cases and more than 5 million deaths worldwide [1]. In an attempt to curtail the spread of the virus, countries across the globe have repeatedly imposed a variety of quarantine strategies, including short-term lockdowns, home curfews, restrictions on social gatherings, cancellations of public events, and travel restrictions [2]. During the initial stages of the pandemic, the focus was primarily on reducing virus transmission and the number of fatalities [3]. Over time, in light of the dramatic alterations to people’s lives caused by both the pandemic and the attendant governmental measures, there has been emergent concern over the consequences of the pandemic for psychosocial functioning. Indeed, a recent systematic review shows that the prevalence of mental health problems among the general population, such as distress, anxiety, and depression, was higher during the pandemic than prior to it (i.e., the overall pooled prevalence was 41.1%, 31.9%, and 31.4%, respectively, in comparison to prevalence rates of 6.2%, 14.7%, and 10.8%, respectively, in pre-pandemic research) [4]. Brooks and colleagues [5] also reported a wide range of negative psychological issues stemming from quarantine, such as stress, confusion, and anger. In addition to the impact on mental health, the pandemic also had profound social and economic consequences, namely, impoverished social lives and unprecedented job losses [6,7].

Although the pandemic affects all segments of society, its consequences are particularly detrimental to groups who were already more reliant on help and support from others prior to COVID-19, including the elderly and people with (intellectual) disabilities [8]. Initially, insufficient research attention was paid to these vulnerable groups when considering the pandemic’s impact [9]. In addition, both groups seemed to be forgotten in the public debate with respect to the imposed COVID-19 restrictions in the Netherlands. In this respect, the elderly and people with intellectual disabilities, including those receiving long-term care, were largely overshadowed by the narrow focus on acute care of COVID-19 patients during the first stages of the pandemic [10], and, indeed, appeared to be an ‘afterthought’ in terms of responses to the pandemic [11,12]. For example, in the Netherlands, which provides the context for the present study, both the elderly and people with intellectual disabilities have been further isolated from society as a result of the restrictive measures that implored ‘vulnerable’ people to stay indoors (e.g., the temporary closure of long-term care facilities, day-care activities, and work services) [13]. Moreover, in some countries, these groups experienced troubling disparities with respect to accessing healthcare services (e.g., intensive care units) due to selective medical triage policies [14,15].

As the pandemic has evolved, however, there has been growing concern over the impact of the COVID-19 pandemic on the elderly and people with intellectual disabilities [16,17]. Studies have shown that they are at greater risk of developing more severe symptoms from COVID-19, which in the case of people with intellectual disabilities stems from common comorbid underlying physical health problems [18,19]. Furthermore, the negative impact on the mental well-being of the elderly and people with intellectual disabilities has become evident. Specifically, research shows that the elderly have reported a decreased quality of life [20], alongside increased levels of loneliness [21], anxiety, depressive and stress symptoms [22], and negative affect [23] during the COVID-19 pandemic. Similarly, studies focused on people with intellectual disabilities have shown that they experienced increased mental health problems during the pandemic, including feelings of anxiety, stress, depression, and loneliness [24,25]. Moreover, research indicates that their social lives have been negatively affected by the pandemic (i.e., reduced social contact) [22,26] and that they have become less active [27,28]. Both the elderly and people with intellectual disabilities have also reported difficulties in finding sufficient help during the pandemic to be able to carry out functional needs [26,29].

These findings provide valuable insight into the impact of COVID-19, especially given that most are from the perspective of the elderly and people with intellectual disabilities themselves (or based on reports by their caregivers). However, this does not necessarily mean that the pandemic’s impact on these groups is seen and/or acknowledged by the general public, organizations, and policymakers. Consequently, in order to be able to sufficiently address the needs and concerns of the elderly and people with intellectual disabilities in the context of COVID-19, it is of paramount importance that the public is also cognizant of how the pandemic has deleteriously impacted these groups. Therefore, the first aim of the present study is to assess the general public’s perceptions of how the COVID-19 pandemic has impacted the elderly and people with intellectual disabilities.

Although this study focused on both the elderly and people with intellectual disabilities, it is important to underscore that it is likely that these groups are perceived differently by the public. One reason for this is the fact that there has been a greater focus on the elderly than there has on people with (intellectual) disabilities in political discussions and public discourse (e.g., media) on the COVID-19 pandemic [15,30]. Moreover, the general public’s perceptions of the impact on the elderly and people with intellectual disabilities may also be influenced by the degree of familiarity with these groups. Previous research has demonstrated that higher levels of familiarity is related to more positive attitudes toward people with intellectual disabilities [31]. Similarly, research has indicated that a higher quality of contact with the elderly is related to more positive attitudes being shown toward them [32]. Hence, people who are more familiar or are in closer contact with the elderly and people with intellectual disabilities (e.g., in their private life or in a work context) may also be more cognizant of how the pandemic has impacted these individuals. Consequently, they may be more likely to sufficiently address their needs and concerns and provide the help and support that they need (e.g., informal care). Based on this, the second aim of the present study is thus to assess the levels of familiarity and contact quality as potential correlates of the perceived impact of the pandemic. 

### The Present Study

The specific aims of this study were to (a) examine the general public’s perceptions of how the COVID-19 pandemic has impacted the elderly and people with intellectual disabilities, including whether there are any potential differences between the perceived impact on these two groups; and (b) study the relationship between the levels of contact quality/familiarity and the general public’s perceptions of how the pandemic has impacted the elderly and people with intellectual disabilities. 

## 2. Methods

### 2.1. Design and Participants 

As part of a broader research project assessing public stigma toward the elderly and people with intellectual disabilities during the COVID-19 pandemic [33], the respondents were recruited via two channels. First, a large online panel provider, MultiScope, distributed a survey among 2300 individuals. Only those respondents that answered content-related questions were included, which resulted in a response rate of around 70% for questions related to the elderly (*n* = 1597) and people with intellectual disabilities (*n* = 1533). Additional respondents were recruited via social media (Facebook, LinkedIn, Twitter), announcements in newsletters, and partner organizations of the [Academic Collaborative Center Living with an intellectual disability (Tranzo, Tilburg University, the Netherlands) removed for blind peer review purposes] (with the initial aim being to follow these respondents over time) (*n* = 250 and *n* = 248, for questions concerning the elderly and people with intellectual disabilities, respectively). Respondents of ≥70 years (*n* = 389) (risk group COVID-19 as per [34]) and people who reported having intellectual disabilities (*n* = 20) were excluded from the corresponding (separate) datasets. In other words, the responses of the elderly related to items targeting this population were not included in the analyses as we wanted to provide the general public’s perceptions of how the COVID-19 pandemic has impacted the elderly; including participants who belong to this population themselves may bias the results. The responses of the elderly related to items targeting people with intellectual disabilities, however, were included in this study, and vice versa. The final datasets for questions related to the elderly and people with intellectual disabilities thus contained 1458 and 1761 individuals, respectively. 

Table 1 provides an overview of the sample characteristics. For both datasets, just over half of the sample were female, while most had completed higher education.

### 2.2. Procedures and Materials 

This study was approved by the Ethics Review Board of [Tilburg University removed for blind peer review purposes] (RP226). An online survey was developed by the research team comprising questions pertaining to demographic characteristics, personal experiences with COVID-19, both the frequency and quality of the contact with the elderly, familiarity with individuals with intellectual disabilities, and the perceived impact of COVID-19 on the elderly and people with intellectual disabilities. The complete survey also included questions related to stereotypes and stigma (the results have been published previously; see Dekker et al.). At the beginning of the online survey, a brief definition of ‘elderly’ was provided (i.e., being 70 years of age or older); in line with previous studies within the general public, a definition of intellectual disabilities was not provided [31]. 

Following a strict lockdown in early 2020, including the closure of schools and all non-essential shops and a ban on visiting the elderly and people with intellectual disabilities residing at group homes, at the time the online survey was active (December 2020–January 2021), the Dutch government was forced to implement restrictions in daily visitors and group sizes (e.g., max two visitors a day at home), the obligation to wear masks in public transport and indoors, and the closure of restaurants and cafés.

**Personal experiences with COVID-19.** The respondents were asked the extent to which the COVID-19 pandemic personally affected them in relation to six situations: being at higher risk of becoming severely ill from COVID-19, either their own or others’ (i.e., a loved one) contamination or (past) hospitalisation, losing their job, and the loss of a loved one. These items were developed by the research team, based on previous questionnaires [35,36] and the literature [21,33]. The response options were “yes” and “no”, with the exception of those questions regarding being at risk (i.e., an additional “I don’t know” option was provided) and contamination (i.e., “me”, “someone close to me”, “both”, or “neither”). Based on the most severe reported experience with COVID-19, six groups were subsequently created (using a ranking system): (1) “no experience with COVID-19” (rank item 0; i.e., all items answered with “no” or “neither”), (2) “being at risk” (rank item 1), (3) “contamination” (rank item 2), (4) “hospitalisation” (rank items 3–4), (5) “losing their job” (rank item 5), and (6) “loss of a loved one” (rank item 6). 

**Contact frequency and quality with the elderly.** To assess the level of familiarity with the elderly, both the frequency and quality of contact were rated (adapted from [32]). The frequency of either face-to-face or online contact with the elderly was assessed using two separate 8-point scales (1 “daily”, 4 “multiple times each month”, 8 “never”). Respondents rated their quality of contact with the elderly on a scale of 1 “very low” to 5 “very high”.

**Familiarity with individuals with intellectual disabilities.** The level of familiarity with individuals with intellectual disabilities was assessed by using the Level of Contact Report [37]. In line with previous research [31], items were adapted to refer to intellectual disabilities. Respondents were asked to check all of the situations that they had experienced (e.g., “I have worked with a person who had an intellectual disability at my place of employment”). The familiarity score was the rank score of the most intimate situation indicated by the respondent. In accordance with Pelleboer-Gunnink et al. [31], the data was merged into four categories: (1) “no familiarity in real life” (rank item 1–4; e.g., watching a documentary), (2) “familiarity in passing by” (rank item 5; observing on a frequent basis), (3) “familiarity at work” (rank item 6–8; e.g., providing treatment), and (4) “familiarity in their private life” (rank item 9–12; e.g., friend of the family). 

**Perceived impact.** Respondents were asked to separately rate their perceptions of the impact of the COVID-19 pandemic on both the elderly and individuals with intellectual disabilities. The items developed for the purposes of this study were based on previous literature investigating the impact of COVID-19 on the elderly [21] and people with intellectual disabilities [25]. Several themes were assessed, including quality of life (1 item), loneliness (1 item), physical and mental health (6 items), quality and frequency of social contact (9 items), activities (7 items), and self-reliance (4 items, reverse coded). All the questions asked the respondents to compare the current situation to the period prior to COVID-19 (e.g., “Can you indicate to what extent you think that “the elderly” undertake the following activities less or more often now as compared to the period before COVID-19?”. For example, “walking or cycling” or “reading”). Items were rated on a scale from 1 (“much less”) to 5 (“much more”); items pertaining to quality of life and quality of social contact were rated on a scale from 1 (“much lower”) to 5 (“much higher”). Mean impact scores were calculated for those themes that contained more than one item (for physical and mental health, while the items relating to feeling anxious, down, and stressed were reverse coded). 

### 2.3. Data Analyses 

Analyses were performed using SPSS Statistics version 24 for Windows. First, descriptive analyses were conducted with respect to the respondents’ personal experiences with COVID-19, frequency and quality of contact with the elderly, familiarity with individuals with intellectual disabilities, and the perceived impact of both the pandemic and the attendant measures (descriptive analyses were conducted separately for questions pertaining to the elderly (using the sample without those ≥70 years of age; *n* = 1458) and individuals with intellectual disabilities (using the sample that excluded those who reported having an intellectual disability; *n* = 1761). Second, to assess the relationship between contact quality with the elderly and the perceived impact (using the sample without those ≥70 years of age; *n* = 1458), hierarchical linear regression models were used. Step 1 included the demographic characteristics that were significantly related to the impact outcome measures (as covariates). In addition, step 2 involved the addition of contact quality. Third, to assess the difference between the four categories of familiarity with people with intellectual disabilities on the impact measures (using the sample that excluded those who reported having an intellectual disability; *n* = 1761), a multivariate analysis of covariance was conducted. Post hoc pairwise comparisons were carried out using Bonferroni correction. Fourth, paired-samples t-tests were conducted to assess potential within-subject differences in the perceived impact on both the elderly and individuals with intellectual disabilities (using the sample that excluded those ≥70 years of age and those who reported having an intellectual disability and who answered all the impact questions with respect to the elderly and individuals with intellectual disabilities; *n* = 1394) (i.e., quality of life, physical and mental health, loneliness, social contact, activities, and self-reliance mean scores). Quality of life and loneliness (ordinal variables with 5-point Likert scale) were treated as continuous impact variables [38].

Prior to analyses, data were checked for normality. Given that the kurtosis and skewness of the variables fell within the range of ±7 and ±2, respectively [39], normality was assumed. There were multivariate outliers on the perceived impact measures (*n* = 47 for questions related to the elderly; *n* = 44 for questions related to people with intellectual disabilities). The full sample was used for all analyses; in the event that removing these outliers yielded significantly different results, both statistics were reported. 

## 3. Results

### 3.1. Descriptive Results

Table 1 presents the descriptive results pertaining to personal experiences with COVID-19, contact with the elderly, and familiarity with people with intellectual disabilities. For both datasets, around half of the respondents reported having neither personal experiences with COVID-19 nor being at increased risk of COVID-19 themselves (categories 1–2; 48.9% for questions related to the elderly; 51.9% for questions related to individuals with intellectual disabilities). Some respondents reported having lost their jobs (4.0% and 3.3%, for the elderly and people with intellectual disabilities, respectively) or losing a loved one (8.2% and 8.3%, for the elderly and people with intellectual disabilities, respectively). Regarding contact with the elderly, 40.1% indicated having either face-to-face or online contact on a weekly basis (one or more times per week). Most respondents were positive about the quality of their contact with the elderly (i.e., high quality; 84.8%). Almost half of the respondents indicated being familiar with people with intellectual disabilities either via work (e.g., providing services/treatment) or in their private life (e.g., a friend of the family) (44.7%; categories 3–4).

Table 2 presents the descriptive results related to the perceived impact on the elderly and individuals with intellectual disabilities. Overall, the quality of life and physical and mental health of the elderly and people with intellectual disabilities was considered to be lower in comparison to before the COVID-19 pandemic (e.g., feeling more anxious, down and stressed; 79.1–90.8% for the elderly; 73.8–76.5% for individuals with intellectual disabilities), while they were both perceived as being lonelier (90.7% and 74.5%, respectively). Both the quality and frequency of social contact for the elderly and individuals with intellectual disabilities (e.g., with family, friends, and neighbours) were deemed to be lower than prior to the pandemic (59.2–88.3% and 46.2–80.2%, respectively), although the frequency of contact with partners and healthcare providers was generally perceived as being the same (48.2–70.0% and 55.1–72.2%, respectively). Both the elderly and individuals with intellectual disabilities were generally regarded as engaging in fewer activities (i.e., shopping, sports, grocery shopping, and (voluntary) work; 71.8–89.2% for the elderly; 67.5–82.6% for individuals with intellectual disabilities), but were believed to be watching television more often (79.6% and 73.2%, respectively). The majority of the respondents perceived the elderly and individuals with intellectual disabilities as being less self-reliant than they were prior to the pandemic, particularly with respect to mental health and maintaining social contact (72.2–75.0% and 61.6–68.0%, respectively).

### 3.2. Contact Quality with the Elderly and the Perceived Impact of the Pandemic on This Group

In this section, the views of the general population are reflected, excluding those ≥70 years of age. Hierarchical linear regressions were conducted to assess whether contact quality with the elderly was significantly related to the impact measures, after controlling for the influence of gender, age, and personal experiences with COVID-19 (i.e., when significantly related to the respective impact measure). There were no significant effects of contact quality on any of the six impact measures. 

### 3.3. Familiarity with People with Intellectual Disabilities and the Perceived Impact of the Pandemic upon this Group

In this section, the views of the general public are reflected, with the exception of those respondents who reported having an intellectual disability. With respect to the questions related to individuals with intellectual disabilities, regression analyses showed a significant multivariate effect of familiarity on the perceived impact measures, *F*(18, 5223) = 4.31, *p* < 0.001, V = 0.044, η^2^ = 0.015, when correcting for gender, age, education, and personal experiences with COVID-19. Significant between-subject effects were found for physical and mental health, *F*(3, 1744) = 6.92, *p* < 0.001, η^2^ = 0.012; social contact, *F*(3, 1744) = 13.80, *p* < 0.001, η^2^ = 0.023; activities, *F*(3, 1744) = 10.11, *p* < 0.001, η^2^ = 0.017; and self-reliance, *F*(3, 1744) = 5.66, *p* = 0.001, η^2^ = 0.010. Pairwise post-hoc Bonferroni comparisons showed that those reporting no familiarity with people with intellectual disabilities in real life perceived their level of physical and mental health to be significantly higher than those reporting familiarity either in passing (*p* = 0.007) or in their private life (*p* < 0.01). They also perceived the quality and frequency of social contact and self-reliance of individuals with intellectual disabilities as being significantly higher than those who reported familiarity in passing (*p* = 0.01; *p* = 0.46), at work (*p* < 0.01; *p* = 0.05), or in their private life (*p* < 0.01; *p* = 0.02). In addition, those reporting no familiarity in real life deemed that people with intellectual disabilities engage in significantly more activities than those reporting familiarity at work or in their private life (*p*’s < 0.01). Similarly, the respondents who reported familiarity in passing perceived people with intellectual disabilities as engaging in more activities than those reporting familiarity at work (*p* = 0.07).

When excluding multivariate outliers on the impact measures (i.e., questions related to intellectual disabilities; *n* = 44), the respondents who reported no familiarity in real life also perceived the physical and mental health of people with intellectual disabilities to be significantly higher than those who reported familiarity at work (*p* = 0.025); those who reported familiarity in passing also perceived the quality and frequency of social contact of people with intellectual disabilities as being significantly higher than those reporting familiarity at work (*p* = 0.047); respondents who reported no familiarity perceived the self-reliance of people with intellectual disabilities as being significantly higher than those reporting familiarity at work (*p* = 0.023) or in their private life (*p* = 0.004)

### 3.4. Differences between the Perceived Impact of the Pandemic on the Elderly and People with Intellectual Disabilities 

This section delineates the views of the general population, excluding those ≥70 years of age and those who reported having an intellectual disability. There were some differences between the perceived impact on the elderly and people with intellectual disabilities. In comparison to before COVID-19, the quality of life of the elderly (*M* = 1.90; *SD* = 0.58) was perceived as being significantly lower than that of individuals with intellectual disabilities (*M* = 2.13; *SD* = 0.57), *t*(1393) = −12.80, *p* < 0.001. The elderly (*M* = 4.28; *SD* = 0.79) were also perceived as being significantly lonelier than individuals with intellectual disabilities (*M* = 3.83; *SD* = 0.71); *t*(1393) = 18.15, *p* < 0.001, in addition to being thought to have significantly lower physical and mental health (*M* = 2.18; *SD* = 0.42) than individuals with intellectual disabilities (*M* = 2.32; *SD* = 0.42); *t*(1393) = −11.72, *p* < 0.001. Similarly, the quality and frequency of elderly people’s social contact (*M* = 2.29; *SD* = 0.50) were perceived as being significantly lower in comparison to that of individuals with intellectual disabilities (*M* = 2.43; *SD* = 0.49); *t*(1393) = −12.01, *p* < 0.001, along with being thought to be less self-reliant (*M* = 2.48; *SD* = 0.53) than individuals with intellectual disabilities (*M* = 2.56; *SD* = 0.51); *t*(1393) = −5.53, *p* < 0.001.

## 4. Discussion

This study examined the general public’s perceptions of how the COVID-19 pandemic impacted the elderly and people with intellectual disabilities as well as the relationship between this perceived impact and levels of familiarity and contact quality. Through administering a cross-sectional survey to a sample of the Dutch population, this study was able to show that the general public is generally aware of the negative impact of the pandemic on the elderly and people with intellectual disabilities. More specifically, both groups were deemed to have a lower quality of life, physical and mental health, and quality and frequency of social contact than they did prior to the COVID-19 pandemic, not to mention being perceived as more lonely and less self-reliant. When comparing the perceived impact of the pandemic on both groups, the impact on the elderly was regarded as being higher than it was for people with intellectual disabilities. Furthermore, those who had no familiarity with people with intellectual disabilities in real life perceived the physical and mental health, quality and frequency of social contact, activities, and self-reliance of these individuals as being higher than those who had greater familiarity, and as such, considered the impact of the pandemic to be smaller. 

Previous studies have shown that the pandemic has had a profoundly negative impact on the elderly and people with intellectual disabilities, namely in terms of their physical health [4,18], psychological well-being [25,40], levels of loneliness [21,25], social lives [22,41], engagement in activities [27,28], and self-reliance [26,29]. In this respect, the findings of the present study testify to the fact that the general public is generally cognizant of this negative impact, albeit with some exceptions. For example, the majority of the respondents perceived the physical health of the elderly and people with intellectual disabilities as being similar to what it had been prior to the pandemic. However, previous studies have shown that both groups are at greater risk of developing more severe symptoms after contracting COVID-19 [18,19]. Furthermore, the frequency of the help required for personal care and housekeeping was generally perceived as being the same during the pandemic as it was before. However, both the elderly and people with intellectual disabilities are often dependent on care and support (e.g., help with performing daily activities), and this support may have been more difficult to find during the pandemic [26,29]. The findings of this study are important insofar as they underscore the need to make the general public more cognizant of this impact; therefore, efforts should be made to increase the public’s awareness of how the pandemic has deleteriously impacted the self-reliance of these vulnerable groups, in order to be able to sufficiently address their specific needs and concerns. Such efforts constitute a vital preliminary step toward a society where values such as compassion and looking out for one another are of paramount importance. To this end, both getting to know and becoming more familiarized with the elderly and people with intellectual disabilities represents a crucial step. Related to this, being aware of the general public’s perceptions is important as it can condition social stigma in pandemic situations. For example, from the moment that the elderly were ‘labelled’ as a population at risk during the COVID-19 pandemic, they felt that people’s perception of them changed. This is in addition to these populations already suffering the intersection of other social stigmas (e.g., ageism and ableism).

Importantly, the pandemic’s impact on the elderly was perceived as being higher than the impact on people with intellectual disabilities. This finding is in line with previous research, which also showed that people are more aware of how the pandemic has negatively impacted the elderly than they are of its impact on people with disabilities [42]. One potential explanation for this result might be the different levels of familiarity with these groups. Generally speaking, prior to COVID-19 people were less familiar with individuals with intellectual disabilities (i.e., around 42% were familiar in the context of their work or private life) [31], not to mention that these individuals were less visible within society, in comparison to the elderly. Indeed, from the start of the pandemic, there has been a significantly greater focus on the elderly in comparison to people with intellectual disabilities, both in contemporary public discourse and political discussions. Specifically, the elderly have been portrayed as a homogeneous group that is, among other things, ‘at risk’, ‘weak’, and ‘vulnerable’ [43], whereas people with intellectual disabilities have been under-represented in contemporary public discourse [30]. This, in turn, could have resulted in people being more aware of the pandemic’s impact on the elderly than they are of its impact on people with intellectual disabilities. Moreover, there is also an observed tendency to trivialize the impact on people with intellectual disabilities, which could be due to prevailing contradictory cognitions; on the one hand, perceiving these individuals as ‘warm’ and ‘friendly’ [44], while on the other, an awareness that they are isolated from society and being discriminated against during COVID-19 (e.g., with respect to access to healthcare) [14]. In such situations of cognitive dissonance, information that does not fit in with prevailing attitudes or beliefs may simply be disregarded [45].

### Limitations and Future Directions 

This study assessed the perceived impact of the pandemic on the elderly and people with intellectual disabilities in general. However, it is important to stress that both groups are highly heterogeneous. For example, the pandemic’s impact may depend on a person’s level of functioning (e.g., mild or profound intellectual disability), while those living in residential facilities may have experienced the pandemic’s impact differently than those living at home as a result of the governmental measures to contain the virus (i.e., not being allowed to leave the care facility, visiting arrangements; [13]). It would be interesting for future research to explore whether the COVID-19 experiences of both the elderly and people with intellectual disabilities living in different settings would be different. In addition, whereas a brief definition of ‘elderly’ was provided, a definition of the term ‘intellectual disabilities’ was not provided to the participants. Respondents based their answers on their own perceptions of people with intellectual disabilities. This provides valuable insight into people’s responses to the plain label ‘intellectual disabilities’; it might be that participants had an incorrect interpretation of the term and confused the term with ‘mental disorder’. Moreover, the measures we used to assess familiarity with people with intellectual disabilities and the elderly were based on previously developed instruments in the respective fields [31,32], which precludes any direct comparison between the levels of familiarity with these two groups. Consequently, future research should seek to develop and use a measure of familiarity that enables such a direct comparison. Related to this, as most of the other measurements used were not previously validated measures, the replicative validity is limited. Furthermore, the findings of this study solely reflect the views of Dutch respondents. The COVID-19 pandemic, however, is a global threat that arguably has had varying impacts on individual countries around the world. Cross-cultural research is therefore needed in order to be able to generalize the findings of this study to different countries. In addition to this cross-cultural research on the elderly and people with intellectual disabilities, it would be interesting to focus on other vulnerable populations, such as children and people with mental disorders. Finally, most of the respondents were recruited via an online panel. The resulting sample was generally representative of the broader Dutch population with respect to gender, but skewed toward more highly educated and older people. In this respect, the findings should be interpreted with caution when attempting to generalize them to the general population.

## 5. Conclusions

This is the first study, at least to the best of our knowledge, that has generated important insights into the general public’s perceptions of how the COVID-19 pandemic has impacted upon the elderly and people with intellectual disabilities. Generally, people were cognizant of the deleterious impact of the pandemic on these particular groups. However, the impact on the elderly was deemed to be higher than the impact on those with intellectual disabilities. Similar to previous research [31], more than a quarter of the sample were unfamiliar with individuals with intellectual disabilities, and it was especially this portion of the sample who appeared to be less cognizant of the pandemic’s impact on these individuals. These findings thus have important implications, namely in terms of increasing the general public’s awareness of the pandemic’s negative impact on these vulnerable groups, which is a necessary precondition for being able to sufficiently address their specific needs and concerns. Finally, the findings also underscore the need for these groups to become more visible within society, via, for instance, self-advocacy, education, and enhanced intergroup contact, which is particularly relevant during these challenging times. That is, for example, it would be important for people with intellectual disabilities to collectively advocate to become visible within society. Hence, there is a great need for sufficient resources, and the relevance of advocacy groups should be widely acknowledged [46]. Healthcare providers for people with intellectual disabilities, healthcare professionals, and public health policy play essential roles in this process. Funding and policy support seem to be essential to empower people with intellectual disabilities as do advocacy groups, to clearly address the specifics needs and concerns of people with intellectual disabilities.

## Figures and Tables

**Table 1:** Sample characteristics.

	*n* = 1458 ^1^		*n* = 1761 ^2^	
Demographic Attribute	*n*	%	*n*	%
Gender				
Male	613	42.0	855	48.6
Female	841	57.7	903	51.3
Other	4	0.3	3	0.2
Age				
18–24 years	45	3.1	42	2.4
25–39 years	313	21.5	296	16.8
40–54 years	555	38.1	528	30.0
55–69 years	545	37.4	528	30.0
70–84 years	-	-	361	20.5
85 years or older	-	-	6	0.3
Education				
Low	161	11.0	259	14.7
Mid	439	30.1	518	29.4
High	855	58.6	980	55.7
None	3	0.2	4	0.2
Ethnicity				
Dutch	1288	88.3	1577	89.6
Migration background	170	11.7	184	10.4
Urbanisation ^a^				
Not urbanised	101	6.9	121	6.9
Hardly urbanised	301	20.6	378	21.5
Moderately urbanised	231	15.8	293	16.6
Strongly urbanised	437	30.0	529	30.0
Extremely urbanised	366	25.1	412	23.4
Missing ^b^	22	1.5	28	1.6
Personal experiences of COVID-19 ^c^				
None	480	32.9	488	27.7
Being at risk	233	16.0	427	24.2
Contamination	479	32.9	544	30.9
Hospitalisation	87	6.0	98	5.6
Loss of job	59	4.0	58	3.3
Loss of loved one	120	8.2	146	8.3
Contact frequency with the elderly ^d^				
Daily	116	7.9	-	-
Weekly	585	40.1	-	-
Monthly	333	22.9	-	-
Yearly or never	424	29.1	-	-
Contact quality with the elderly				
Low	26	1.8	-	-
Moderate	195	13.4	-	-
High	1237	84.8	-	-
Familiarity with people with intellectual disabilities ^e^				
No familiarity in real life	-	-	492	27.9
Familiarity in passing	-	-	481	27.3
Familiarity at work	-	-	255	14.5
Familiarity in private life	-	-	533	30.3

^1^ For questions related to the elderly. ^2^ For questions related to individuals with intellectual disabilities. ^a^ Urbanisation based on the surrounding address density of a neighbourhood, as per Statistics Netherlands. ^b^ Treated listwise. ^c^ Based on the respondents’ most severe experience with COVID-19. Contamination and hospitalisation refer to self (in the past) or a loved one. ^d^ The *n* and percentages refer to the average amount of face-to-face and online contact with the elderly. ^e^ The *n* refers to the number of times that the respondents rated items within this category as their most intimate contact with individuals with intellectual disabilities.

**Table 2:** Descriptives of the perceived impact of COVID-19 on the elderly and individuals with intellectual disabilities.

	Elderly (*n* = 1458)				Intellectual Disabilities (*n* = 1761)			
Item	*M (SD)*	Less (%)	The Same (%)	More (%)	*M (SD)*	Less (%)	The Same (%)	More (%)
**Quality of life**	1.90 (0.59)	89.6	9.5	1.0	2.16 (0.57)	76.0	23.5	0.6
**Physical and mental health**								
Anxious †	4.02 (0.53)	0.8	9.9	89.4	3.82 (0.58)	1.2	23.3	75.4
Down †	4.10 (0.55)	0.4	8.8	90.8	3.83 (0.56)	0.8	22.7	76.5
Stressed †	3.89 (0.59)	1.0	19.9	79.1	3.82 (0.60)	1.0	25.3	73.8
Happy	2.15 (0.62)	78.3	19.3	2.4	2.41 (0.60)	59.1	38.3	2.6
Peaceful	2.38 (0.80)	62.8	28.5	8.7	2.37 (0.70)	63.7	30.4	5.9
Healthy	2.56 (0.65)	44.2	52.5	3.3	2.77 (0.49)	24.3	73.7	2.0
**Loneliness**	4.29 (0.80)	3.4	6.0	90.7	3.80 (0.72)	4.5	21.0	74.5
**Social contact**								
Quality	1.84 (0.70)	88.3	9.2	2.5	2.09 (0.59)	80.2	18.6	1.2
*Frequency:*								
Partner	3.01 (0.68)	14.6	70.0	15.4	2.90 (0.60)	18.3	72.2	9.5
Parents	-	-	-	-	2.67 (0.84)	46.2	36.9	16.9
Children	2.39 (0.86)	65.6	20.2	14.1	2.54 (0.74)	49.3	42.2	8.5
Other family	1.94 (0.84)	80.2	13.6	6.2	2.14 (0.76)	74.5	20.3	5.2
Friends and acquaintances	1.88 (0.79)	83.3	12.6	4.1	2.06 (0.75)	77.7	18.0	4.3
Neighbours	2.39 (0.81)	59.2	31.9	8.9	2.29 (0.71)	64.5	31.2	4.3
Shop assistants	2.00 (0.74)	77.3	20.4	2.3	2.05 (0.68)	76.5	22.3	1.2
Healthcare providers	2.86 (0.81)	31.7	48.2	20.1	3.03 (0.75)	20.6	55.1	24.3
**Activities**								
Walking or cycling	2.74 (0.99)	44.0	31.0	25.0	2.62 (0.78)	45.9	41.5	12.6
Reading	3.70 (0.67)	3.1	30.0	66.9	3.16 (0.66)	10.7	61.9	27.4
Watching television	3.93 (0.64)	1.4	18.9	79.6	3.83 (0.63)	1.2	25.6	73.2
(Voluntary) work	1.81 (0.69)	86.5	12.3	1.2	2.16 (0.70)	70.1	28.3	1.6
Grocery shopping	2.18 (0.68)	71.8	25.8	2.4	2.23 (0.65)	67.5	31.2	1.4
Shopping	1.76 (0.67)	89.2	9.7	1.2	1.94 (0.67)	82.6	16.4	1.0
Sports	1.94 (0.70)	82.9	15.2	1.9	2.10 (0.67)	75.1	23.5	1.4
**Self-reliance** ††								
Maintaining social contact	2.24 (0.75)	72.2	22.2	5.6	2.39 (0.73)	61.6	31.7	6.7
Mental health	2.22 (0.75)	75.0	18.3	6.7	2.30 (0.74)	68.0	25.2	6.8
Personal care	2.82 (0.59)	21.7	72.9	5.4	2.86 (0.51)	17.8	77.7	4.5
Housekeeping	2.65 (0.68)	37.2	57.1	5.8	2.77 (0.60)	28.2	66.0	5.8

Note. Emboldened items indicate composite variables or items used separately in the analyses (i.e., quality of life, loneliness). † Scores were reversed for calculation of the mean score. †† Scores were reversed, so that a higher score reflects more self-reliance. For quality of life and quality of social contact, percentages relate to “lower”, “the same”, and “higher” scores.

## Data Availability

The data that support the findings of this study are, on the basis of a Data Transfer Agreement and in consultation with the Ethics Review Board of Tilburg University, available from the corresponding author upon reasonable request.
